# Nurse-led diabetic retinopathy screening: a pilot study to evaluate a new approach to vision care for Canadian Aboriginal peoples

**DOI:** 10.1080/22423982.2017.1422670

**Published:** 2018-01-31

**Authors:** Shelley Spurr, Carol Bullin, Jill Bally, Krista Trinder, Shahab Khan

**Affiliations:** ^a^ College of Nursing, University of Saskatchewan, Saskatoon, Canada; ^b^ College of Medicine, University of Saskatchewan, Saskatoon, Canada; ^c^ Prince Albert, Canada

**Keywords:** Aboriginal, type 2 diabetes, retinopathy, nurse-led vision care, Canadian

## Abstract

Diabetic retinopathy is the most common cause of new cases of blindness and is pandemic among Aboriginal people around the world. To reduce health inequities, accessible vision screening among these high-risk populations is essential. To assess cardio-metabolic co-morbidities associated with type 2 diabetes and the use of a portable fundus camera as a novel approach for convenient, earlier and more accessible vision screening for Aboriginal peoples living with type 2 diabetes in northern and remote Canadian communities. This quantitative pilot study screened participants diagnosed with type 2 diabetes for commonly associated cardio-metabolic co-morbidities using anthropometrical measurements, blood pressure and a A1c (HbA1c) blood glucose test, followed by vision exams conducted first by a trained nurse and then by an ophthalmologist to screen for signs of retinopathy using fundus photography. Large numbers of the participants presented with overweight/obese (84.8%), pre-hypertension/hypertension (69.7%) and an elevated A1C (78.8%). Inter-rater reliability demonstrated substantial agreement between vision exam judgements made by the nurse and ophthalmologist (k = .67). Nurse-led vision screening in remote or northern communities can improve the standard of care by extending access to health services, lowering the costs to families by reducing travel expenses and preventing vision loss in a family member.

## Background and purpose

Aboriginal people living in Canada are among the highest risk populations for diabetes and related complications, including retinopathy [1]. Diabetic retinopathy is the most common cause of new cases of blindness in adults ages 20–74 [2,3]. Nearly two-thirds (60%) of people living with type 2 diabetes for 20 years or more suffer from retinopathy, and many individuals (21%) will have symptoms of retinopathy at the time their diabetes is diagnosed [2]. The increasing prevalence of diabetes and the associated complications in Aboriginal peoples illustrate the pressing need to understand the impact of this chronic disease.

To date, visual health has been largely ignored, which has resulted in significant numbers of Aboriginal Canadians suffering from serious ocular and visual health problems. Only a few ocular health programmes have evolved targeting Aboriginal communities [4]. An extensive literature review indicated that Aboriginal people living in remote communities are at high risk for developing type 2 diabetes and that many remain undiagnosed and at risk for the associated complications, including vision loss and blindness [5–11]. In addition, evidence suggests northern and remote communities experience significant barriers to accessing health care, notably visual health [4]. This reality supports the need to develop a unique pathway for vision care in these northern communities. As such, the purpose of this pilot study was to investigate the cardio-metabolic co-morbidities of type 2 diabetes and the possibility of using a portable fundus camera (Optovue) as a novel approach for convenient, earlier and more accessible vision screening and referral for people living with type 2 diabetes in northern and remote Canadian, predominantly Aboriginal communities. This mobile state-of-the-art technology allows for quick vision screening and can be used by registered nurses (RNs) to screen for visual changes related to diabetes. The data can be saved and any images of concern securely emailed to an off-site ophthalmologist for further investigation.

A few studies have evaluated the ability of other health professionals to screen for diabetic retinopathy screening [12,13]. For example, a team from Australia travelled to 11 remote communities to conduct fundus screening. The images were forwarded to and analysed by a general practitioner who was accredited to perform diabetic retinopathy identification and grading. Findings from this study found that this model of care significantly improved access to diabetic retinopathy screening in remote communities [12]. Similarly, in Flinders Australia, a study examined the quality assurance of using an ophthalmic nurse practitioner (NP) with special training in ophthalmic disease to screen patients for diabetic retinopathy in a local ophthalmology clinic. Results showed a very high concordance between the findings of the ophthalmologist and the trained NP [13]. However, this article reports new and unique findings from a population-based investigation into the cardio-metabolic co-morbidities of type 2 diabetes and the possibility of RNs using a portable fundus camera (Optovue) as a novel approach for vision screening of Aboriginal peoples living with type 2 diabetes in northern and remote Canadian communities. To our knowledge, this is the first study examining the possibility of RN-led diabetic retinopathy screening in Canadian Aboriginal peoples. In Canada, Aboriginal Peoples is a collective name for all original peoples and their descendants, including First Nations (status and non-status Indians), Inuit and Métis peoples [14], and is used as a general term in this article.

## Methods and procedures

Ethical approval from the University of Saskatchewan Research Ethics Board was obtained. All participants were informed of their right to withdraw from the study or to refuse to partake in any part of the study. Prior to data collection, a written informed consent was used to explain the purpose and procedures, right to withdraw, possible risks and benefits and confidentiality of the study. Participants were reassured that participation, or withdrawal, from the study would not affect their care from the clinic from which they were recruited. Finally, the participants were instructed to call the research nurses if they had any questions or to request the results of the study.

### Study design

This quantitative pilot study screened participants diagnosed with type 2 diabetes for commonly associated cardio-metabolic co-morbidities using anthropometrical measurements, blood pressure and a haemoglobin A1C (HbA1c) blood glucose test, followed by vision exams conducted first by a registered nurse and then by an ophthalmologist to screen for signs of retinopathy using fundus photography.

### Population

A purposeful sample of adults living in northern rural and remote Canadian, predominantly Aboriginal communities, were invited to participate in this pilot study. The participants (n=33) were recruited from an ophthalmology clinic in a northern Canadian city and met the following qualifying criteria: (a) >18 years old and (b) diagnosed with type 2 diabetes. Although some participants lived in the city where data collection took place, most travelled from rural and remote communities to access the northern urban ophthalmology clinic. Participant results were excluded if they were previously diagnosed with other vision co-morbidities.

### Co-morbidity measurements

The co-morbidities associated with type 2 diabetes were measured as recommended by the Canadian Diabetes Association (2013) and included screening for weight, height, body mass index (BMI), blood pressure, and HbA1c. Standard procedures were used to measure weight and height. Using weight and height values, BMI was calculated and used to classify participants as normal weight, overweight or obese according to the Canadian guidelines for body weight classification in adults [15]. As such, overweight and obesity were classified as a BMI of 25–29.9 and ≥30, respectively.

Blood pressure was included as a metabolic co-morbidity and was measured according to the National Institute of Health guidelines [16], which defines 4 blood pressure risk categories: (1) normal (systolic <120 and diastolic <80); (2) pre-hypertension (systolic = 120–130 or diastolic = 80–89); (3) hypertension stage 1 (systolic = 140–159 or diastolic = 90–99) and (4) hypertension stage 2 (systolic = 160 or higher or diastolic = 100 or higher).

HbA1c was used as a diagnostic tool to measure glycaemic control [17]. An HbA1c “A1C Now+”™ point-of-care assay provided quantitative measurement of the % of glycated haemoglobin over the last 3 months. The average level of blood glucose in the 30 days immediately preceding the blood sample contributes ~50% of the HbA1c results whereas ~10% is from the past 90–120 days [18,19]. The accuracy of the A1C Now+ was tested on participants who were diabetic and non-diabetic (n=189) in a multiple-site study conducted in the USA, with a 99% accuracy rate found when compared to the National Glycohemoglobin Standardization Program reference results [20]. The present study identified 2 categories of HbA1c: normal (7% or lower) and elevated (7.1% or higher).

Ethnicity (Aboriginal and non-Aboriginal), age and gender are also identified as co-morbidities by the Canada Diabetes Association [7]. Therefore, screening included obtaining such demographic data from the participants.

#### Data collection

Two nurses completed the assessments in a private room within the ophthalmology clinic, taking approximately 15 min in all. A research assistant distributed and collect prepackaged consent forms to the participants prior to the assessment.

#### Analysis

Descriptive statistics were computed using the Statistical Package for Social Sciences (v.22.0). Further, chi-square analyses were conducted to investigate if the risk factors of hypertension and obesity occurred at higher frequencies for males and females who presented with an increased HbA1c level.

### Vision exams

Prior to data collection, one nurse was trained by a technologist employed in the ophthalmologist clinic to obtain fundal images of clients using a portable fundus camera (Optovue). Data from this training period were excluded from the present study.

Immediately following the co-morbidity screening, participants in the present study also took part in vision screening. A single drop dilation protocol (approximately 10 min to dilation) with 1 drop each of tropicamide and phenylephrine was used for all participants. The first vision exam involved the Optovue-trained nurse screening for retinopathy using a portable fundus camera. The second eye exam involved fundus photography with a Zeiss Viscam500 camera that was completed by the ophthalmologist. The nurse-led vision exam results were compared to those of the ophthalmologist to determine the accuracy of the vision assessment for detection of diabetic retinopathy. Concordance was assessed by a simple yes/no response which determined if diabetic retinopathy was present or absent.

#### Analysis

Cohen’s kappa was used to evaluate inter-rater agreement between the nurse and ophthalmologist. The kappa value measured agreement with the assessment of diabetic retinopathy being present or absent (yes/no).

## Results

A total of 33 participants were screened for diabetic cardio-metabolic co-morbidities and retinopathy. The sample had a similar representation of male (N = 17) and female (N = 16) participants. All of the female participants and 65% of the male participants self-identified as Aboriginal. The participants ranged in age from 29 to 74 with an average of 56 years. Demographic data are reported in Table 1.

**Table 1.** Demographics.

	Male (N = 17)		Female (N = 16)	
Variable	N	%	N	%
Ethnicity				
Aboriginal	11	65	16	100
Non-Aboriginal	6	35	0	0
Age				
Under 30	1	6	0	0
30–39	3	18	2	13
40–49	0	0	3	19
50–59	6	35	4	25
60–69	6	35	6	38
70+	1	6	1	6
Years of diabetes				
<5	3	18	3	20
5–10	3	18	4	27
11–15	3	18	3	20
16–20	3	18	5	33
>20	5	28	0	0

### BMI

BMI was calculated using participant height and weight data and ranged from 18.75 to 48.63 with a mean of 32.22 (SD = 6.88). Weight classification is reported in Table 2. The percentages of obese (BMI ≥ 30) and overweight (BMI 25.0–29.9) participants were 54.6% and 30.3%, respectively. Overall, 88% of men and 82% of women were classified as either obese or overweight. Although the males had a higher prevalence of overweight than females (35% vs. 25%) but lower obesity prevalence (53% vs. 57%), these differences were not statistically significant.

**Figure 1.**
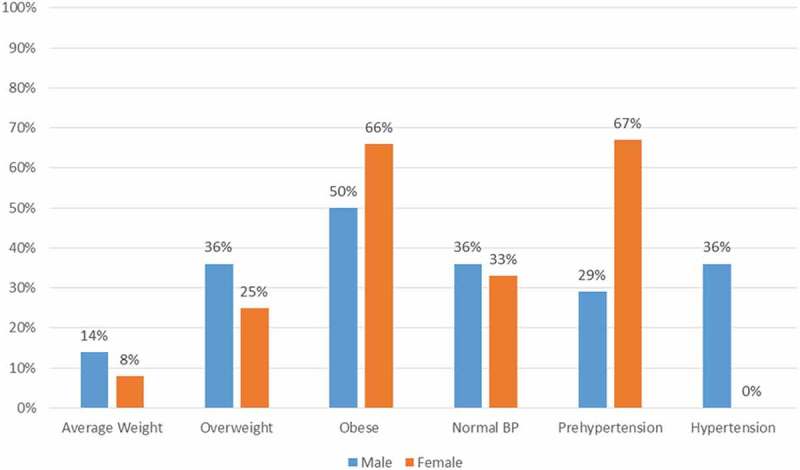
Weight and blood pressure classification for those with elevated A1C by sex.

### A1c

HbA1c levels ranged from 5.50 to 13.00 with a mean of 8.30 (SD = 1.64). The overall percentage of participants with elevated HbA1c levels (7.1 or higher) was 78.8%; percentages by sex are reported in Table 2. No statistically significant differences were found between male participants (82%) and female participants (75%) with increased HbA1c levels.

**Table 2.** Weight, A1C and blood pressure classification by sex.

Variable	Male, N = 17		Female, N = 16	
Weight classification	N	%	N	%
Average weight	2	12	3	19
Overweight	6	35	4	25
Obese	9	53	9	57
A1C classification				
Normal	3	18	4	25
Increased	14	82	12	75
Blood pressure				
Normal	5	29	5	31
Pre-hypertension	5	29	9	56
Hypertension	7	41	2	13

### Blood pressure

The percentage of participants with pre-hypertension (systolic = 120–130 or diastolic = 80–89) or hypertension (systolic = 140–159 or diastolic = 90–99) was 69.6%. Percentages by sex are reported in Table 2. Similar numbers of males (70%) and females (69%) had pre-hypertension/hypertension.

### Blood pressure and weight classification for participants with an elevated A1C level

Further analysis showed that the participants with an elevated A1C were also overweight/obese (89%) and pre-hypertensive/hypertensive (65%). A gender difference analysis concluded that 91% of females and 86% of males were overweight/obese. Also notable from this analysis is the large number of females (67%) and males (65%) with pre-hypertension/hypertension (Figure 1). Specifically, men with increased A1C levels were significantly more likely to have hypertension than women (p=0.042).

### Vision screening

**Figure 2.**
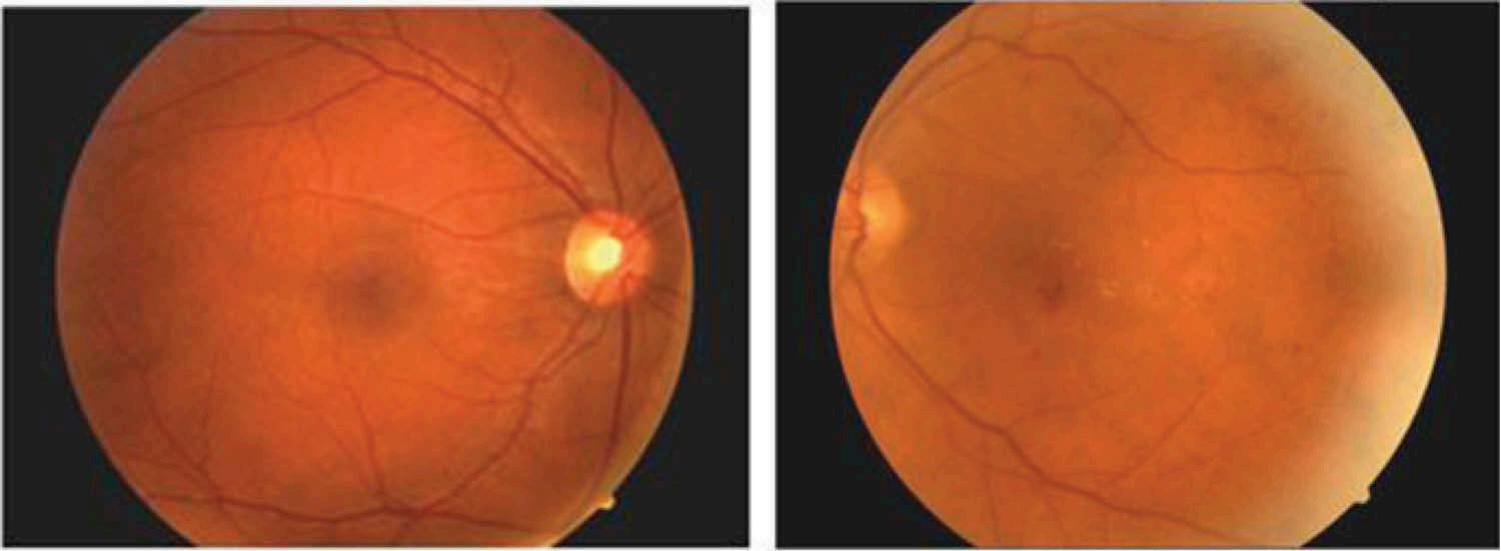
Diabetic retinapathy.

Inter-rater reliability was calculated using Cohen’s kappa, with results demonstrating a substantial agreement between the health professionals’ judgements (k = .67). Using fundal photography, the nurses were able to assess and identify diabetic retinopathy (Figure 2). The overall percentage of participants with retinopathy was 83.3%. No statistically significant differences were found between male and female participants. Results comparing the assessment of diabetic retinopathy between the RN and physician are presented in Table 3.

**Table 3.** Retinopathy as assessed by a physician and nurse.

	Yes		No	
Rater	N	%	N	%
MD	25	83	5	17
RN	24	80	6	20

## Discussion

Evidence suggests that Aboriginal people with diabetes experience disparities in complications and mortality [21]. The higher rates of adverse outcomes are associated with a number of factors including lifestyle [1]. Specifically, the increased prevalence of overweight and obesity in Aboriginal people is a significant factor contributing to the cardiovascular co-morbidities associated with type 2 diabetes and is a serious health concern [22]. For example, one study conducted in a northern Canadian community found the proportion of overweight/obese was 84.4% [23]. A recent systematic review found that, among adult Aboriginal populations in Canada, 66.3% were overweight/obese; males had higher overweight prevalence than females (34% vs. 26.6%) but lower obesity prevalence (31.6% vs. 40.6%) [24]. Wharton et al. [22] found that 80–90% of persons with type 2 diabetes were overweight/obese and that a higher BMI was associated with increased mortality. Aboriginal people are also burdened with higher rates of cardio-metabolic risk factors such as hypertension, which may indicate future risk for cardiovascular morbidity and mortality [21]. Findings from the current study confirm this trend and are disconcerting because these cardiovascular co-morbidities left untreated can lead to heart attack, stroke and kidney damage [22]. As such, the validation of these results illustrates the need for renewed vigilance to develop a wide range of community-led, culturally relevant health promotion and primary prevention activities for Aboriginal peoples [25].

Lifestyle modifications are recommended for type 2 diabetes, including strict glycaemic control to slow the development and progression of complications, including retinopathy [2]. One large study conducted in northern Canada revealed that 61.1% of the participants had HbA1c levels >7, indicating that most were not achieving glycaemic targets [26]. Similar results were found in the present study and were also disconcerting considering Aboriginal peoples with diabetes experience serious disparities in diabetes-related complications and mortality than the general population with type 2 diabetes, including chronic kidney disease, amputations and macrovascular disease, all of which limit a person’s quality of life [26–28].

Furthermore, vision loss due to retinopathy is a serious complication of diabetes and is associated with significant morbidity including increased falls, hip fractures and a fourfold increase in mortality [2,29]. A large American study conducted in the largest county in Los Angeles evaluated the use of teleretinal diabetic retinopathy screening (with optometrists conducting the analysis) and found improved efficiency, quality and access to care [30]. A smaller scale study conducted in Australia evaluated the effectiveness of a team travelling to conduct diabetic retinopathy screening in remote communities. In this case, a general practitioner identified and graded the diabetic retinopathy [12]. Other telemedicine programmes have been developed to identify and refer clients with diabetic retinopathy in Canada and other countries [2,13]. These models of care improved eye care for those with limited access. However, many Aboriginal Canadians suffer from serious visual health problems and this remains an issue that has largely gone ignored [4]. Fragmented health care, poor chronic disease management, high health care staff turnover and limited or non-existent surveillance have contributed to higher rates of adverse health outcomes for Aboriginal people [31]. Other researchers argue that the predominant individual-focused approach and blaming of the individual has not worked to improve diabetes management in this population [32]. Because northern primary health care is delivered in Canada overwhelmingly by RNs, this reality supports the need to developing a unique pathway for vision care in these northern and predominantly Aboriginal communities. One study found that expanding the scope of practice for RNs in diabetes care was an effective strategy for blood pressure management in Aboriginal people living in remote and rural communities where doctors were scarce [33]. To our knowledge, there are neither RN-led fundus screening programmes in northern Canadian communities nor investigations into the potential method of use [2]. Thus, the present study whereby the RNs conducted the fundus eye screening and accurately analysed the visual images (r=.67) is a new and unique finding. In addition, the vision assessment by a physician using a Zeiss camera agreed well with the RN’s use of the optovue camera, which makes the study more impactful in terms of error avoidance.

Nurse-led fundus photography is a distinct and exciting opportunity to provide vision screening and assessments in rural, remote or northern communities and extends access to standard care for vulnerable populations at risk for retinopathy due to type 2 diabetes. Health care relationships are central to addressing the ongoing colonial dynamics that are contributing to the increased rates of chronic disease and to the health inequities for Aboriginal people [34]. RNs working closely with patients and families are well positioned to build positive relationships by taking a genuine interest in the clients and their personal circumstances, showing empathy and patience. Additionally, vision care from a trusted nurse can provide renewed confidence in the health care system, potentially removing the barriers to diabetes care for Aboriginal people [34]. As such, these research findings build capacity for addressing the co-morbidities associated with type 2 diabetes by having nurses empowered with the knowledge and skills required to develop local solutions that are adapted to the realities and culture of Aboriginal peoples; in particular, this study provides a potential solution to the disparate ocular health challenges experienced in remote and northern communities.

This research has the potential to contribute to significant economic and social outcomes. The immediate outcome of implementing nurse-led retinopathy screening is the early detection of eye disease for people living in remote and northern communities. Second, these findings illustrate the importance and practice of nurse-led diabetes screening and vision care, leading to long-term sustainability in addressing this issue through the northern nursing workforce. Finally, these results demonstrate the usefulness of nurse-led fundal screening as a tool to improve the accessibility and affordability of vision care in the north.

### Limitations

Although a small sample was used to conduct this pilot study, participants were from predominantly northern and remote Aboriginal communities in a Western Canadian province which allowed for a wide sample that was representative of Aboriginal peoples. The findings of this study confirm the trends with respect to the prevalence of overweight/obesity and pre-hypertension/hypertension in Aboriginal peoples diagnosed with type 2 diabetes, and therefore appear congruent with other Canadian studies investigating co-morbidities of type 2 diabetes in this population [22,24]. To validate these results, duplicating this research with a larger sample and subsequent research designs is indicated. A second limitation was the potential for statistical bias as there was a relatively high rate of retinopathy present and the results may be somewhat different with a lower rate of retinopathy in the study population. A third limitation includes the time factor for the nurses to learn fundal photography. In the present study, a technologist taught the nurse how to use the Optovue camera for the screening process. Once this 1-day learning session was complete, the nurse felt comfortable using the camera and was able to complete the vision screening in a timely fashion. The final limitation of the study is that participants’ eyes must be dilated prior to the assessment. As such, the nurse would be required to dilate the pupils prior to the assessment. Although the process is simple, a standard physician order is required to complete this task.

## Conclusion

RN-led vision screening in remote or northern communities can improve the standard of care by extending access to health services, lower the costs to families by reducing travel expenses and prevent loss of vision for a member of a family. This research is being used as a foundation to advocate for expanded primary health services to improve the health outcomes for Aboriginal people living with diabetes in remote and northern communities.
